# Viability of Molds and Bacteria in Tempeh Processed with Supercritical Carbon Dioxides during Storage

**DOI:** 10.1155/2018/8591015

**Published:** 2018-10-01

**Authors:** Maria Erna Kustyawati, Filli Pratama, Daniel Saputra, Agus Wijaya

**Affiliations:** ^1^Department of Agriculture Product Technology, University of Lampung, Bandar Lampung 34145, Indonesia; ^2^Department of Agriculture Technology, University of Sriwijaya, Palembang 30662, Indonesia

## Abstract

Application of supercritical carbon dioxide for processing of food products has an impact on microbial inactivation and food quality. This technique is used to preserve tempeh due to no heat involved. The quality of tempeh is highly influenced by mold growth because of its role in forming a compact texture, white color, and functional properties as well as consumer acceptance. This study aims to observe viability of molds and bacteria in tempeh after processed with supercritical CO_2_ and to determine the best processing conditions which can maintain mold growth and reduce the number of bacteria in tempeh. For that purpose, tempeh was treated using high pressure CO_2_ at 7.6 MPa (supercritical CO_2_) and at 6.3 MPa (sub/near supercritical CO_2_) with incubation period of 5, 10, 15, and 20 min. The best treatment obtained was used to process tempeh for storage study. The results showed that there was a significant interaction between pressure and incubation period for bacterial and mold viability at *ρ*>0.05. Reduction of bacteria and molds increased with longer incubation period. Molds were undetectable after treatment for 20 min with either supercritical CO_2_ or sub-supercritical, and bacteria significantly reduced up to 2.40 log CFU/g. On the other hand, sub-supercritical CO_2_ for 10 min was the best processing method because molds survived 4.3x10^4^ CFU/gram after treatment and were able to grow during storage at 30°C, producing white mycelium as indicated by increasing the *L*⁎ color value and tempeh acceptability. The inactivation of mold was reversible causing it to grow back during storage under suitable conditions. Tempeh matrix composition can provide protection against the destructive effects of supercritical CO_2_. Gram-positive bacteria were more resistant than Gram-negative. In conclusion, sub-supercritical CO_2_ can act as a method of cold pasteurization of tempeh and can be used as an alternative method to preserve tempeh.

## 1. Introduction

Consumer needs for food are not only in terms of health and food safety but also food with minimal processing that can maintain the quality of freshness and taste for a certain length of storage. Thermal food preservation is an effective technique for reducing microbial count of foods. However, for heat sensitive food products it can give undesirable sensorial changes and destroy the nutritional quality of the food. High pressure carbon dioxide technology is a nonthermal alternative processing to improve the microbial safety of the product while preserving nutritional and sensorial characteristics. It is known that carbon dioxide under the supercritical phase (7.4 M.Pa and 31.06°C) has unique properties. Carbon dioxide has dual characteristics where it is like a gas with high diffusivity and a liquid with high solubility which enable it to easily diffuse through complex matrices and extract substances “Liao [1]”. The supercritical CO_2_ (scCO_2_) characteristic has expanded its use for the inactivation of various vegetative microorganisms in food as a nonthermal technology without loss of taste, color, and nutrients “Calvo and Torres [2]”. Sub-supercritical carbon dioxides (sub-scCO_2_) treatment causes microbial inactivation and can avoid changes in sensory attributes of food quality. In relation to microbial growth and food processing, “Garcia-Gonzales [3]” and “Guo [4]” found that carbon dioxide can stimulate and inhibit cellular development, where inhibitory measures have been used to improve the hygiene of liquid and solid food by inactivating bacterial growth. The study conducted by “Kustyawati [5]” found that processing with supercritical CO_2_ and sub-supercritical CO_2_ retained the texture, vitamin B, Ca, and protein content, but reduced fat, water content, and some volatile compounds in tempeh.

Tempeh is generally sold in fresh form, even though it is not consumed in raw state, but needs to be processed further before consuming. Tempeh is a fermented soybean product by* Rhizopus oligosporus*, but bacteria and yeasts are also involved during the fermentation and contributed significantly to the production of functional metabolites. The microbial community structure in tempeh is a very important feature in maintaining not only the sensory appearance but also the functional nature of the tempeh. Supercritical carbon dioxide technology can be an alternative process for tempeh which is expected to reduce the number of bacteria and at the same time maintain high mold growth. The high number of bacteria in tempeh can interfere with mold growth and consequently the tempeh will spoil more quickly. Molds growth is needed to produce tempeh with a compact texture, white gray color, and being palatable. Previous research has shown that sub-supercritical CO_2_ at 6.3 MPa for 10 min did not significantly affect tempeh color and the tempeh was acceptable “Kustyawati [5]”. However, the survival of microorganisms in tempeh processed with supercritical CO_2_ has not been revealed. Minimal processing technology without involving heat that can maintain the growth of mold in tempeh is needed in an effort to increase the shelf-life and maintain the freshness of tempeh, nutritional value, and consumer preferences. The aim of this study was to observe viability of molds and bacteria in tempeh after processed with supercritical CO_2_ and determine best processing which can reduce bacteria but maintain mold life and to observe the ability of the mold to grow during storage.

## 2. Materials and Methods

### 2.1. Processing of Tempeh

**Figure 1:**
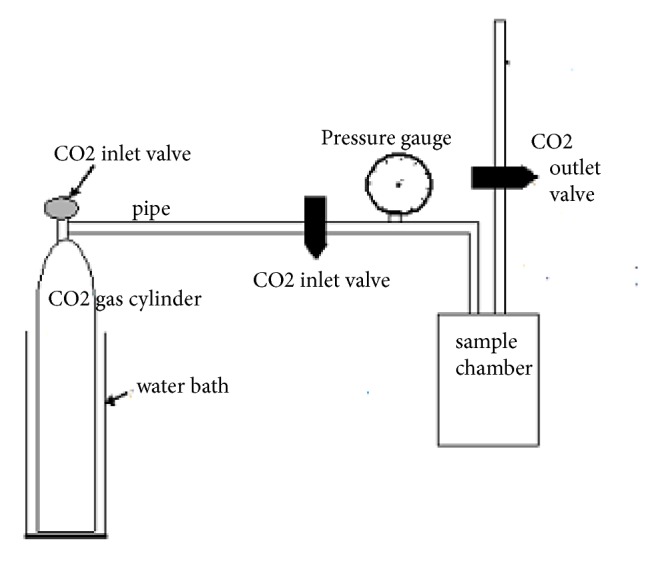
The diagram of the experimental apparatus.

The high pressure CO_2_ installation used for experimental treatments consists of a CO_2_ gas cylinder, a cylindrical pressure chamber, pressure gauges, and a water bath at constant temperature “Saputra [6]” (see Figure 1). Tempeh, in the form of cylinder with 3.5cm in diameter and 10 cm in length, was obtained from the Center of Home Industry Tempeh Making Palembang, Indonesia. Fresh tempeh was placed in a pressure chamber and then closed tightly. When the designated temperature in water bath was reached and all pipe connections were secured, commercially available CO_2_ (PERTAMINA, Jakarta, Indonesia) was injected through the gas inlet valve from the gas cylinder into the pressure chamber until it reached the desired pressures of 6.3 and 7.6 MPa (showed in pressure gauge) within 1 min. After being subjected to high pressure CO_2_ treatment for the specified incubation period, the pressure was lowered to atmospheric pressure within 3 minutes by slowly opening the gas outlet valve. Then the tempeh was aseptically removed from the pressure chamber using a sterilized tong, placed in the sterilized container, and stored in a refrigerator before conducting the analysis such as SEM, but the samples were directly analyzed for microorganism analysis.

The experiment was conducted in a full factorial design with the factors as follows: supercritical CO_2_ (scCO_2_) treatment at 7.6 MPa for 5, 10, 15, and 20 min and sub-supercritical CO_2_ (sub-scCO_2_) treatment at 6.3 MPa for 5, 10, 15, and 20 min. Each treatment was replicated three times.

### 2.2. Enumeration of Bacteria and Molds

The tempeh was plated no later than 1 hour after the processing. Samples (5 g) were homogenized (1:3) with phosphate buffer solution (BPS) in a Stomacher 400 for 1 min, and appropriate dilutions of the homogenate were made. The enumeration of the bacteria in the Nutrient agar (NA Difco, USA) and molds in the potatoes dextrose agar (PDA, Difco USA) plates was done after incubation period 32°C for 24 h for bacterial and of 27°C for 4 days for molds counts. Oxytetracycline 0.05% and chloramphenicol 0.05% were added to the media to inhibit the growth of bacteria, and cyclohexemide 0.05% was added to inhibit the growth of yeasts. Results were reported as log CFU/g for each treatment (CFU, colony forming units). The degree of inactivation was determined by evaluating the log (N/N0) versus time, where N0 (CFU/g) was the number of microorganisms initially present in the unprocessed sample and N (CFU/g) was the number of survivors after the processing.

The surviving bacteria after supercritical CO_2_ processing were isolated and identified by the PCR sequencing analysis. The isolated strains were identified based on morphological characteristics, the biochemical profile according to the manufacturer's instructions (API system, Biomerieux, France), and sequencing 16S (bacteria)* r*DNA as described below.

### 2.3. Analysis of DNA

The work of bacterial identification was done according to the method developed by “Parton [7]” as followed. To each of the isolates three to four colonies were picked up and suspended into 100 *μ*L of sterile ddH_2_O (double-distilled water). Extraction was done by heating at 100°C for 5 min to lyse the cells and centrifuged at 13.000 g for 15 min at 4°C. The supernatant which is containing DNA was transferred to an Eppendorf tube. Two *μ*L of each DNA sample was used as a template in the Polymerase Chain Reaction assay. The primers 355F (5'-CCT ACG GGA GGC AGC AG-3') and 910r (5' –CCC GTC AAT TCC GAG TT– 3') were used for bacterial cells. A final 50 *μ*L volume was used containing 5 *μ*L of forward primer 10 *μ*m (Sigma), 5 *μ*L of reverse primer 10 *μ*m (Sigma), 2 *μ*L of template DNA, 5 *μ*L of 10x Taq DNA polymerase buffer (Sigma), 4 *μ*L MgCl_2_ (25 mm), and 0.5 *μ*L Taq DNA polymerase (5 u/*μ*L, Sigma). The PCR conditions applied for bacteria were 95°C for 1min, followed by 30 cycles at 95°C for 30 sec, 50°C for 1 min, and 72°C for 1 min, followed by one final extension at 72°C for 6 min. The Microcon PCR columns (Millipore, CA, USA) were used to purify the amplicons, and the purified products were eluted with 35 *μ*L of Milli-Q sterile water. PCR sequencing reaction, which was of eight* ng* of the DNA, was performed as follows: 3.2 *μ*L of forward primer (1*μ*M), 6 *μ*L of Big Dye Buffer (Applied Bio Systems), 2 *μ*L of Big Dye Mix (Terminator RR Mix, Applied Bio Systems), and Milli-Q sterile water up to 20 *μ*L. The PCR conditions were followed as the one previously described. The extracted DNA was treated with 45 *μ*L of pure ethanol of 4°C and 3.75 *μ*L of EDTA of 125 *μ*M, incubated in the dark for 15 min, and centrifuged at 15,000 g for 15 min at 4°C. The pellet obtained was washed with 150 *μ*L of 70% ethanol and again centrifuged at 15,000 g for 15min at 4°C. The pellets were dried for 15 min at 37°C and supernatants were discharged. The samples obtained were then suspended into the 15 *μ*L formamide and sequencing was carried out at an ABI PRISM 310 Genetic Analyzer (Perkin Elmer). For the identification, the sequences obtained were searched against and compared to those present in the National Center for Biotechnology Information (NCBI) genome bank. The similarity was determined by the percentage of similarity greater than 98.5%.

### 2.4. Storage Study

The optimal condition of the process that was found in the experiment was used to treat the tempeh that would be used for storage study. Tempeh processed at the optimal process condition was stored at 20 and 30°C for 5 days, together with the unprocessed tempeh. During the storage, the total of molds and* L∗* color were analyzed daily. A storage time of 5 days was chosen considering that the shelf-life of fresh tempeh is normally around 1-2 days at room temperatures (30±2°C), while processing of sub-scCO_2_ was expected to extend the shelf-life of the tempeh.

### 2.5. Color Measurement

The surface color analysis of processed and unprocessed tempeh was evaluated as CIE* L∗a∗b∗* value and* LCH* color scale using color difference meter (TC-1500, Tokyo, Japan). Results were expressed as* L∗* (Lightness),* a∗* (redness), and* b∗* (yellowness). The* L∗*,* a∗*, and* b∗* values represent the means of the three measurements for each sample. The total color difference (Δ*E∗*) between the control and the treated tempeh was obtained using the following equation: $\Delta E^{*}=\sqrt{{\Delta L*}^{2}+{\Delta a*}^{2}+{\Delta b*}^{2}\;}\;$where the Δ*L∗*, Δ*a∗*, and Δ*b∗* values meant the difference between the* L∗, a∗*, and* b∗* values after the treatment and the* L∗, a∗*, and* b∗* values of the standard color. The standard color used in this experiment was the* L∗, a∗*, and* b∗* values of the tempeh control.

### 2.6. Scanning Electron Microscope (SEM)

Analysis microstructure of tempeh mycelium microstructure was conducted by scanning electron microscope (SEM JEOL JSM 5310 LV) following “Hong and Pyun [8]” procedures adjusted to tempeh sample. Sample preparation procedures before being observed with SEM were as follows: (1) tempeh was cut according to the* stub* size and affixed to the top of the* stub*, (2) then tempeh was coated with gold by using* IB2* ion coater tool for 5 minutes with ions current of 6-8 miliAmpere, and (3) finally tempeh was observed with ACC 20kV-voltage devices at 2000x, 3000x, and 10000x magnification.

### 2.7. Statistical Analysis

Statistically significant differences (*ρ < 0.05*) between the two types of treatment were determined using analysis of variance (ANOVA) and Duncan's multiple range tests “Gomez and Gomez [9]”.

## 3. Results and Discussion

### 3.1. Bacterial and Mold Inactivation

**Figure 2:**
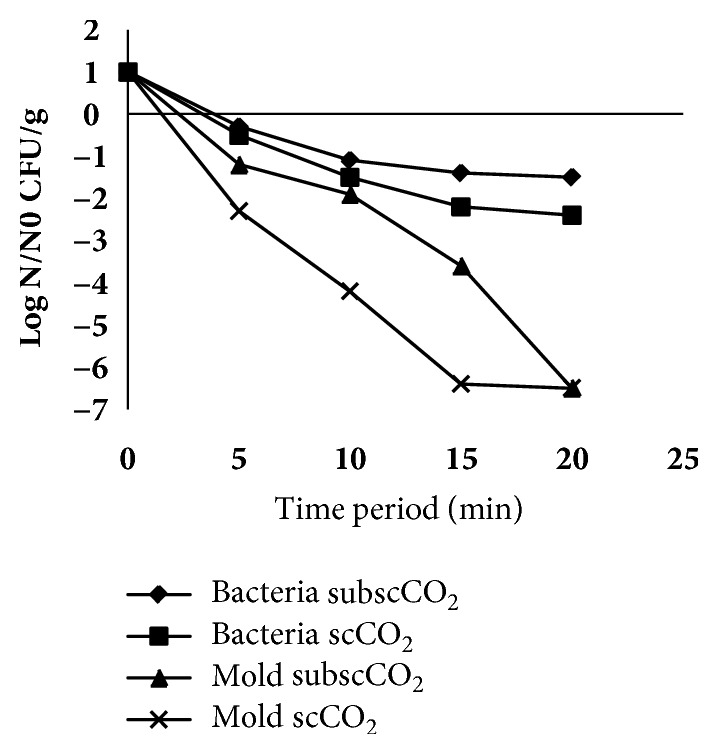
The effect of supercritical (7.6 MPa) and sub-supercritical CO_2_ (6.3 MPa) processing on bacterial and mold inactivation.

The study showed that there was a significant interaction with pressure and holding time for bacteria and molds inactivation at *ρ*>0.05 (see Table 1). The initial bacterial and the mold counts were 2.3x10^7^CFU/g and 6,1x10^6^ CFU/g, respectively. The number of bacteria and molds decreased with increasing pressure and time period applied. Molds were more affected than bacteria (Figure 2). Bacterial number decreased to about 1.7 logs at supercritical CO_2_ and 1.08 logs at sub-supercritical CO_2_ while molds decreased to about 4.88 logs at supercritical CO_2_ and 3.73 logs at sub-supercritical CO_2_. It is suggestive that the inactivation process was not caused by the pressure of pressurized CO_2_. Inactivation process occurred at either sub/near supercritical CO_2_ or supercritical CO_2_. In some studies such as Kimchi processing, the inactivation of microorganisms was achieved at very high CO_2_ pressures, namely, 600 MPa, compared to the inactivation process only at the pressure of 6.3 and 7.6 MPa in this study. The decrease in the number of microorganisms at elevated pressurized CO_2_ can be explained as high solvating power of supercritical CO_2_ extract vital constituents from the cells or cell membranes, resulting in death of the cell. In this case, pressurized CO_2_ penetrates into the cells to build up the density within the cells and expand the cell wall, then it removes intracellular constituent including phospholipids and hydrophobic compounds when the pressure is suddenly released. The removals of constituents alter the structure of the membranes and the balance of the biological system promoting inactivation.

Figure 2 showed that bacteria decreased up to 2.4 logs at supercritical (7.6 MPa) CO_2_ for 20 min, whereas they decreased to 1.5 logs at sub-supercritical (6.3 MPa) CO_2_ for 20 min. In contrast to our study, “Ferrentino [10]” was reported that the optimal conditions to obtain about 3.0, 1.6, and 2.5 log (CFU/g) reductions of mesophilic aerobic, psychrophilic, and lactic acid bacteria in cubed cooked ham were scCO_2_ processing at 12 MPa, 50°C, 5 min. “Gunes [11]” demonstrated that supercritical CO_2_ at 8 MPa can be an effective nonthermal alternative process for pasteurization of grape juice and tomato paste. The inactivation process of microorganisms in tempeh increased in relation to the increase of incubation period of pressurized CO_2_ (see Table 1). Increase in incubation period from 5 to 20 min showed a significant increase in the inactivation values of bacteria and molds where bacterial population decreased from 0.42 to 1.95 logs and molds decreased from 1.75 to 6.5 logs. The explanation to this was that mass transfer rate of CO_2_ was greater with the longer incubation period of the pressure of pressurized CO_2_. At longer incubation period the amount of CO_2_ increases, accumulates into the lipophilic inner layer, and dissolves into and forms hydrogen bond with phospholipid “Mulakhudair [12]”, resulting in destruction to cell structure and function due to breakdown of lipid chains. This will further increase the permeability of cell membrane, making it easier for CO_2_ to enter the cytoplasm cell. In the cytoplasm CO_2_ binds to water and forms HCO_3_^−^ ions, lowering the cytosolic pH which interferes with cell metabolic processes and results in cell death “Garcia-Gonzales [13]”.

**Figure 3:**
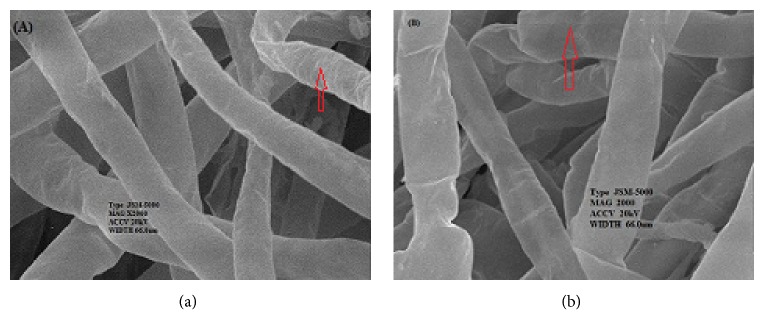
The changes of hyphae mycelium. (a) Hyphae were elastically rigid before the inactivation process at 6.3MPa for 10 min. (b) Hyphae were inflated after the inactivation process at 6.3Mpa for 10 min.

At the increased pressure and time, molds inactivation increased (see Figure 2). Molds decreased to an undetectable number (log 6) for 20 min at sub-supercritical CO_2_ (6.3 MPa) and 15 min at supercritical CO_2_ (7.6 MPa). However, it was found that the countable numbers of molds were 4.3x10^4^ CFU/g at the tempeh processed with sub/near supercritical CO_2_ for 10 min, indicating processing under these conditions is optimal to be applied to tempeh because there are still a number of fungal growths on the surface of tempeh at 10^4^ CFU / g. This is supported by the fact that the mycelium was inflated (see Figure 3(b)). Hyphae mycelium (tempeh control) without inactivation process showed an elastic-rigid texture while mycelium after the inactivation process at 6.3 MPa for 10 min showed that hyphae was inflated (see Figure 3(a)). The minimum number of molds is 10^3^ CFU / g for tempeh to have a compact structure, produce grayish white color, have a functional role, and be accepted by consumers “Kustyawati”.

This finding was in contrast with published data by “Shon and Lee [14]” where molds remained relatively constant in* Kimchi* after treatment up to 600 MPa. Other finding showed that yeasts and molds were undetected in herbs dried with supercritical CO_2_ at 10 MPa for 150 min “Zambon [15]” which was in agreement with our results. The type and chemical contents of products processed with the supercritical CO_2_ could be the reason for differences in findings. During high pressure processing in this experiment, CO_2_ diffused easily into the tempeh matrix because the tempeh contains soluble proteins, fat, carbohydrates, and other polar compounds. The interaction between CO_2_ and matrix macromolecules caused changes in the matrix structure, providing protection for microorganisms in tempeh. In addition, the penetration of CO_2_ into the hyphae caused the cell to be inflated which was reversible due to its elastically rigid texture, comprising a double layer of glycoprotein, glucan, chitin, and melanin “Madigan [16]”.

The number and type of microorganisms present in tempeh depend on the inoculums used in fermentation and fermentation process conditions. Tempeh is a fresh food with water content ranging from 65 to 65.7% (dry weight) “Kustyawati [5]”. When tempeh is processed with supercritical CO_2_, water in contact with pressurized CO_2_ becomes acidic due to the formation and dissociation of H_2_CO_3_ which liberates H^+^ ions, resulting in lowering pH in the tempeh (pH of extracellular). Even though this low pH may diminish resistance to inactivation of microorganisms, the reduction in pH is not enough to cause the lethal effect of CO_2_ on some bacteria in the tempeh. Therefore in this experiment,* Bacillus subtilis, Lactobacillus *sp.*, Pediococcus *sp., and* Streptococcus *sp. were found and isolated from tempeh after processed with pressurized CO_2_ with the pressure of 7.8 MPa for 20 min. In addition to these bacteria,* Klebsiella pneumonia, Citrobacter freundii*, and* Enterobacter cloacae* were also found from tempeh without processing. This finding was in agreement with the published data “Mathias [17]” that supercritical CO_2_ exposure to* Bacillus subtillis* ranging from 2 to 25 MPa did not influence its inactivation. Another study reported that application of high pressure CO_2_ ranging from 200 to 600 MPa resulted in more than 99.99% of cells which were sublethally injured “Ulmer [18]”. Lowered pH of the tempeh may contribute to an increase in cell permeability which facilitates penetration of CO_2_ into microbial cell and accumulates in the cytoplasmic interior of bacterial cell. Cell walls of Gram-negative bacteria are composed of lipopolysaccharide on the outside and a thin layer of peptidoglycan in the inside. Supercritical CO_2_ has hydrophobic properties, which can penetrate the cell wall, and dissolves lipopolysaccharide layer. If too much amount of dissolved CO_2_ enters the cytoplasm, the cell may be unable to maintain the pH homeostasis and pH of the internal cell will begin to decrease to coincide with disruption of cellular activity and result in the cell death “Dillows [19]”. This likely is the reasons of the lethal effect on Gram-negative bacteria in tempeh, whereas it is possible that the resistance of Gram-positive bacteria is due to high impermeability of their cell membrane owing to the thick layers rich of peptidoglycan and basic protein, and thin layer of phospholipid-content, resulting in the limited penetration of CO_2_.

### 3.2. Storage Study

**Figure 4:**
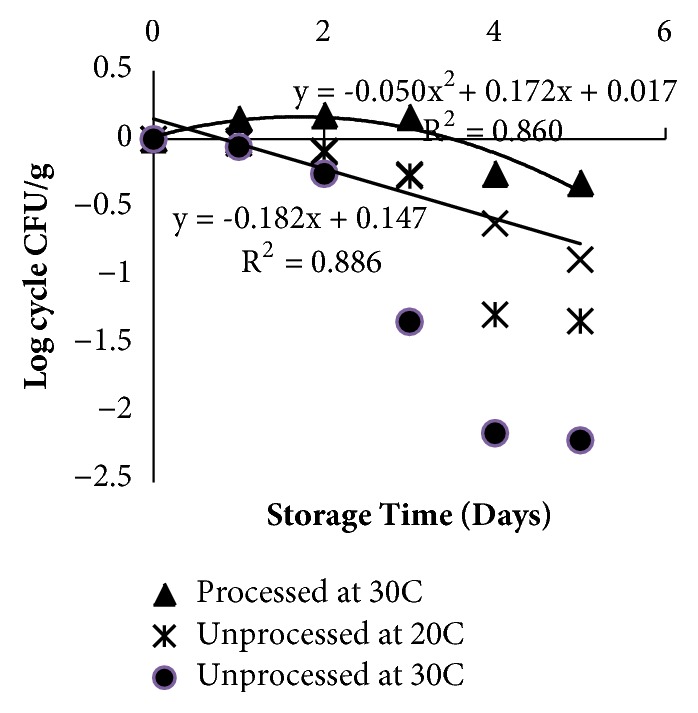
The relationship between mold abundance and storage time at 20 and 30°C of processed tempeh.

It was found that the best processing condition was sub/near supercritical CO_2_ for 10 min. For the storage study, tempeh was processed with sub-supercritical CO_2_ for 10 min and then stored at temperature of 20°C and 30°C. The initial number of molds was 2.5x10^6^ CFU/g before storage and slightly increased during 3 days of storage at 30°C. Relationship between countable mold and storage time showed that the molds increased at a storage temperature of 30°C but decreased at a temperature of 20°C (see Figure 4). High CO_2_ concentration increases the acidity of the medium because CO_2_ reacts with water in the tempeh matrix and produces carbonic acid. Carbonic acid is a weak acid which dissociates to produce H ^+^ ions so that the acidity of tempeh (pH of the tempeh in this study was 5.9-6.1) is favored by molds for their growth, beside optimal growth of mold is at 30°C. This may explain why mold can grow during the storage process.

**Figure 5:**
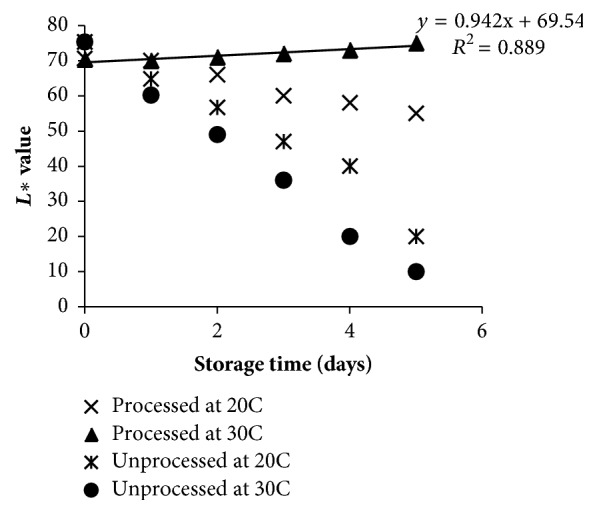
Effect of sub-supercritical CO_2_ on the change of lightness (*L∗*) during storage at 20 and 30°C.

Fresh tempeh has bright white color produced by the growth of mold,* Rhizopus oligosporus*. The brightness of color in tempeh is measured using* L∗* value. Tempeh which has a brownish yellow color indicates that the tempeh has been spoilage, and tempeh that has a dark color produced by spores shows that the tempeh undergoes overfermentation. The color changes in tempeh are caused by, for example, damage to the mycelium of* R. oligosporus*, increased concentration of soy color in a particular area, occurrence of other reactions in tempeh, and spore formations. The* L∗* color kinetic value was showed in Figure 5. High* L∗* (lightness) values showed the whitest bright color of fresh tempeh, while low* L∗* value showed the dark brown color of spoilage tempeh. Compared with unprocessed tempeh,* L∗* color of the processed tempeh showed a slight increase during storage. The* L∗* value of processed tempeh increased after day 1 at the level of 0.94 while it slightly decreased on storage of 20°C at the -3.42 level. Meanwhile, the* L∗* value of unprocessed tempeh showed a rapid decline from the first day of storage at the level of -10.3 to -14.0. An increase of* L∗* value indicated that there was a growing mold. Similar results were reported by “Ferrentino [20]” who observed a significant reduction in color lightness and redness for untreated samples of cloudy apple juice while samples treated with supercritical CO_2_ appeared to have a smaller change when compared with untreated ones. ”Kincal [21]” provides that orange juice treated with a continuous high pressure carbon dioxides (HPCD) system has higher lightness and yellowness when compared with untreated samples during storage.

## 4. Conclusions

Tempeh was used as a model for food product processed with supercritical CO_2_. Ratio of survivor microorganisms (bacteria and molds) in tempeh after each treatment was calculated after spread plating the bacteria and molds in nutrient agar and potatoes dextrose agar plates, respectively. There was a significant interaction with the pressure and incubation period for bacterial and molds reduction at *ρ*>0.05. Reduction of bacteria and molds increased with longer incubation time. Reduction of bacteria 1.5 log was achieved after treatment with supercritical CO_2_ for 10 min and sub-supercritical CO_2_ for 20 min, while mold reduced 6.0 logs after treatment for 20 min with either supercritical or sub-supercritical CO_2_. The longer incubation period may influence microbial reduction in tempeh. Composition of tempeh matrix may give protection against destructive effect of supercritical CO_2_. Gram-negative bacteria in tempeh were dying but Gram-positive bacteria were more resistant to supercritical CO_2_. The inactivation of mold was reversible causing it to grow back during storage under suitable conditions. Therefore, processing with sub/near supercritical CO_2_ for 10 min was the best method to apply to tempeh because molds survived up to 4.3x10^4^ CFU/g and bacteria reduced 1.1 logs, and tempeh is still acceptable to consumer. The treatment can act as a method of cold pasteurization of tempeh and can be an alternative method to preserve tempeh.

## Figures and Tables

**Table 1:** Interaction of high pressure CO_2_ processing and time period on the inactivation of bacteria and molds in tempeh.

Treatments: pressures/time periods	Bacterial reduction log N/No	Mold reduction log N/No
scCO_2_ /5min	0.54±0.52^b^	2,33±0.15^c^
scCO_2_ /10min	1.54±0.22^e^	4,23±0.02^d^
scCO_2_ /15min	2.34±0.12^f^	6,47±0.11^f^
scCO_2_ /20min	2.40±0.13^f^	6,53±0.52^f^
sub scCO_2_ /5min	0.30±0.12^a^	1,17±0.12^a^
sub scCO_2_ /10min	1.10±0.13^c^	1,93±0.20^b^
sub scCO_2_ /15min	1.40±0.11^d^	5,27±0.07^e^
sub scCO_2_/20min	1.50±0.23^e^	6,47±0.41^f^

Note: the numbers in the column followed by the same letter were not significantly different under* p 0.05.*

## Data Availability

The data used to support the findings of this study are available from the corresponding author upon request.
